# Editorial: Epigenetic aspects of autoimmune diseases

**DOI:** 10.3389/fcell.2022.991693

**Published:** 2022-08-11

**Authors:** Wesley H. Brooks, Marina I. Arleevskaya, Yves Renaudineau

**Affiliations:** ^1^ Department of Chemistry, University of South Florida, Tampa, FL, United States; ^2^ Central Research Laboratory, Kazan State Medical Academy, Kazan, Russia; ^3^ INFINITY, Institut Toulousain des Maladies Infectieuses et Inflammatoires, Toulouse, France; ^4^ Laboratoire d'Immunologie, Institut Fédératif de Biologie, Centre Hospitalier Universitaire deToulouse, Université Toulouse III, Toulouse, France

**Keywords:** epigenetics, lupus (SLE), multiple sclerosis, rheumatoid arthritis, sjogren’s syndrome

Genetics, environmental factors, and epigenetics all contribute to autoimmune disease onset and progression (Gulati and Brunner, 2018). Most of the earlier research on autoimmune diseases focused on genetics and environmental factors. Research into genetics, in attempting to identify specific risk alleles has had limited success, for example, identifying HLA-DRB1, a complex of genes coding for cell surface proteins, has emerged as a risk allele for autoimmune diseases such as systemic lupus erythematosus (SLE) and rheumatoid arthritis (RA) (Niu et al., 2015). However, genetic findings have not been sufficient to explain the majority of RA and SLE cases, autoimmune diseases in general, or the typical delay in initial onset, occurring later in life (early to mid-adulthood), in most autoimmune diseases. Even with identification of a risk allele, it is not clear if it is a cause of the disease or just a subsidiary factor. Likewise, environmental factors (e.g., viruses, toxins, bacteria, etc.) also present confusion as to their role since a variety of environmental factors and combinations can be involved in triggering onset of an autoimmune disease (Arleevskaya et al., 2016; Arleevskaya et al., 2020). For example, Epstein-Barr virus (EBV) is suspected of a role in autoimmune diseases, especially multiple sclerosis (MS) (Bjornevik et al., 2022). However, almost all adults have had exposure to EBV but only a small percentage develop MS and onset can be many years after initial infection with EBV. It may be that another environmental factor and/or a genetic risk allele needs to be involved, such as an especially heavy cellular viral load of EBV that may occur by viral binding and entry using HLA-DR cell surface proteins as opposed to other HLA types (Agostini et al., 2018).

Research into the involvement of epigenetics in autoimmune diseases has been steadily increasing in the past two decades. Epigenetics is the control of gene expression or suppression without changing the underlying DNA sequence of the gene (Renaudineau et al., 2011). Epigenetics can involve methylation of DNA, which typically suppresses the underlying gene, or demethylation of the DNA as a step towards expression (Fali et al., 2014). Coordinated with the DNA methylation state are modifications to histone residues based on the “histone code” that further suppresses or opens the gene by altering how tightly the DNA is held by nucleosomes. In addition, non-coding RNA transcripts (i.e., they do not code for a protein) can add to epigenetic control, such as the X Inactivation Specific Transcript (XIST) RNA involved in silencing of the extra X chromosomes in female cells (Bost et al., 2022). Such RNAs can add structural support to heterochromatin and/or recruit the enzymes that modify the DNA and histones.

In this Research Topic, we have gathered articles discussing epigenetics in a variety of autoimmune diseases. Due to autoimmune tautology (i.e., common characteristics among different autoimmune diseases) as described previously by Anaya, we believe autoimmune researchers focused on one disease can learn from the insights and findings of researchers working on other autoimmune diseases (Anaya, 2012; Anaya, 2017; Arvaniti et al., 2019). Li and colleagues present an overview of DNA methylation by discussing its involvement in development of immune cells and in autoimmune diseases. They also provide discussion of aberrant DNA methylation modifications discovered in important disease-related cell types. With regards to specific diseases, Kabeerdoss and colleagues discuss DNA methylation in Takayasu Arteritis, an autoimmune vasculitis of the aorta. In addition, Charras and colleagues discuss a correlation between DNA methylation patterns in CD8^+^ T-cells and clinically active psoriasis in Psoriatic Arthritis, and Vecellio and colleagues present insights into DNA methylation patterns in monozygotic twins discordant for in psoriatic diseases.

RNA methylation and numerous other RNA modifications are important epigenetic biomarkers of interest in relation to normal cellular functions and diseases. Such modifications are especially important in T cell maturation and can impact RNA localization, translation, and alternate splicing among other functions (Chao et al., 2021). Wang and colleagues present a discussion of the m6A RNA methylation with regards to autoimmune diseases and the immune system. Lv and colleagues discuss current understanding of RNA methylation in systemic lupus erythematosus, and Yao and colleagues present expression profiles of mRNAs and long non-coding RNAs in Graves’ disease.

## References

[B1] Agostini S. Mancuso R. Guerini F. R. D’Alfonso S. Agliardi C. Hernis A. (2018). HLA alleles modulate EBV viral load in multiple sclerosis. J. Transl. Med. 16, 80. PMID: 29587799. 10.1186/s12967-018-1450-6 29587799 PMC5870171

[B2] Anaya J. M. (2012). Common mechanisms of autoimmune diseases (the autoimmune tautology). Autoimmun. Rev. 11, 781–784. PMID: 22353402. 10.1016/j.autrev.2012.02.002 22353402

[B3] Anaya J. M. (2017). The autoimmune tautology. A summary of evidence. Jt. Bone Spine 84, 251–253. PMID: 28017820. 10.1016/j.jbspin.2016.11.012 28017820

[B4] Arleevskaya M. I. Kravtsova O. A. Lemerle J. Renaudineau Y. Tsibulkin A. P. (2016). How rheumatoid arthritis can result from provocation of the immune system by microorganisms and viruses. Front. Microbiol. 17, 1296. PMID: 27582741. 10.3389/fmicb.2016.01296 PMC4987382 27582741

[B5] Arleevskaya M. I. Larionova R. V. Brooks W. H. Bettacchioli E. Renaudineau Y. (2020). Toll-like receptors, infections, and rheumatoid arthritis. Clin. Rev. Allergy Immunol. 58, 172–181. 10.1007/s12016-019-08742-z 31144208

[B6] Arvaniti P. Zachou K. Lyberopoulou A. Gatselis N. K. Brooks W. H. Dalekos G. N. (2019). Epigenetic modifications in generalized autoimmune epithelitis: Sjögren's syndrome and primary biliary cholangitis. Epigenomes 3, 15. 10.3390/epigenomes3030015 34968227 PMC8594719

[B7] Bjornevik K. Cortese M. Healy B. C. Kuhle J. Mina M. J. Leng Y. (2022). Longitudinal analysis reveals high prevalence of Epstein-Barr virus associated with multiple sclerosis. Science 375, 296–301. 10.1126/science.abj8222 35025605

[B8] Bost C. Arleevskaya M. I. Brooks W. H. Plaza S. Guery J. C. Renaudineau Y. (2022). Long non-coding RNA Xist contribution in systemic lupus erythematosus and rheumatoid arthritis. Clin. Immunol. 236, 108937. 35114365. 10.1016/j.clim.2022.108937 35114365

[B9] Chao Y. Li H. B. Zhou J. (2021). Multiple functions of RNA methylation in T cells: A review. Front. Immunol. 12, 627455. 10.3389/fimmu.2021.627455 33912158 PMC8071866

[B10] Fali T. Le Dantec C. Thabet Y. Jousse S. Hanrotel C. Youinou P. (2014). DNA methylation modulates HRES1/p28 expression in B cells from patients with lupus. Autoimmunity 47, 265–271. 10.3109/08916934.2013.826207 24117194 PMC4034120

[B11] Gulati G. Brunner H. I. (2018). Environmental triggers in systemic lupus erythematosus. Semin. Arthritis Rheum. 47, 710–717. PMID: 20053532. 10.1016/j.semarthrit.2017.10.001 29169635

[B12] Niu Z. Zhang P. Tong Y. (2015). Value of HLA-DR genotype in systemic lupus erythematosus and lupus nephritis: a meta-analysis. Int. J. Rheum. Dis. 18, 17–28. 10.1111/1756-185X.12528 25546242

[B13] Renaudineau Y. Beauvillard D. Padelli M. Brooks W. H. Youinou P. (2011). Epigenetic alterations and autoimmune disease. J. Dev. Orig. Health Dis. 2, 258–264. 10.1017/S2040174411000353 25141262

